# Toward Fully Bio-Based Polyurethane Foams: Effects of Radish Seed and Tall Oil Polyols on Biofoam Properties

**DOI:** 10.3390/ma18245692

**Published:** 2025-12-18

**Authors:** Mikelis Kirpluks, Maria Kurańska, Elżbieta Malewska, Łukasz Bonder, Nanija Dambe, Dominika Grucela, Stanisław Kuciel

**Affiliations:** 1 Polymer Laboratory, Latvian State Institute of Wood Chemistry, Str. Dzerbenes 27, LV-1006 Riga, Latvia; 2 TENAX PANEL Ltd., str. Spodribas 1, LV-3701 Dobele, Latvia; 3 Faculty of Chemical Engineering and Technology, Cracow University of Technology, Warszawska 24, 31-155 Kraków, Poland; elzbieta.malewska@pk.edu.pl (E.M.);; 4 Faculty of Chemical Engineering and Technology, CUT Doctoral School, Cracow University of Technology, Warszawska 24, 31-155 Cracow, Poland; lukasz.bonder@doktorant.pk.edu.pl; 5 Faculty of Materials Science and Applied Chemistry, Riga Technical University, Str. P. Valdena 3/7, LV-1048 Riga, Latvia; 6 Faculty of Materials Engineering and Physics, Cracow University of Technology, al. Jana Pawła II, 31-864 Kraków, Poland

**Keywords:** bio-based polyurethane foams, radish seed oil, tall oil polyol, open-cell structure, green chemistry, renewable materials, sustainable polymers

## Abstract

The development of bio-based polyurethane foams has become a key direction in polymer materials research, driven by the need to replace petrochemical raw materials with renewable alternatives. This study investigates the synthesis and characterization of open-cell polyurethane foams produced using mixed bio-polyols derived from radish seed oil and tall oil in various mass ratios. For comparison, reference foams based on a radish seed oil polyol, tall oil-based polyol and a petrochemical polyol were also prepared. The influence of the polyol composition on the foaming behavior, cell structure, apparent density, mechanical properties, and thermal conductivity of the resulting foams was analyzed.

## 1. Introduction

Polyurethanes (PURs) are versatile polymers synthesized through the reaction between polyols and isocyanates in various mass ratios. Depending on the desired properties, raw materials with different chemical structures are used together with catalytic systems and various additives such as silicones, flame retardants, blowing agents, adhesion promoters, and fillers [1,2]. Due to their highly adjustable composition and broad spectrum of physical and chemical properties, PURs rank among the most widely used polymeric materials globally, finding applications across numerous industrial sectors.

Rigid PUR foams are mainly used in the construction industry as efficient thermal insulators, while flexible foams are lighter, air-permeable, resistant to permanent deformation, and exhibit good tensile strength. Depending on the formulation and processing conditions, foam density can vary widely—in some cases reaching values below 10 kg/m^3^, making these materials extremely lightweight [3]. A particularly fast-growing segment of the polyurethane industry is spray foams, available in both open- and closed-cell structures. These foams account for approximately 25% of the polyurethane insulation market and represent the fastest-growing PUR product group. In 2024, the global market value for polyurethane insulation materials exceeded United States Dollar (USD) 9 billion, with an expected annual growth rate of 5.5% until 2033 [4,5]. The total market for insulation materials reached USD 55 billion in 2021, with a similar projected growth rate of about 5.4%. The spray polyurethane foam segment alone is expected to grow from USD 2.9 billion in 2024 to USD 5.1 billion by 2032, corresponding to an annual growth rate of 5.6% [6,7]. The overall global polyurethane market was valued at USD 78.1 billion in 2023 and is forecasted to expand at a compound annual growth rate of 3.9% through 2030.

Polyurethane adhesives are widely used in the production of mineral wool sandwich panels due to their excellent bonding strength, thermal stability, and ease of processing [8]. These two-component systems, consisting of a polyol blend and an isocyanate, react to form a durable polymer network that effectively bonds the mineral wool core to metal facings [9]. The adhesive foams during curing, allowing it to penetrate the mineral wool surface and create strong mechanical interlocking. Proper control of reaction kinetics—such as cream, gel, and tack-free times—is essential to ensure uniform adhesion during panel pressing. PU adhesives also provide good resistance to heat, moisture, and mechanical stress, making them suitable for structural and insulating applications in building envelopes [10]. The next step to increase the sustainability of mineral wool adhesives would be to develop a bio-based polyurethane foam with excellent foamability properties.

The broad range of functional properties and high physical–chemical stability make PURs suitable for an ever-expanding spectrum of applications. However, one of their most important advantages remains their exceptionally low thermal conductivity, which ensures outstanding insulation performance at minimal material density and with simple application methods. With the growing global emphasis on sustainable development—particularly within the construction sector—high-performance insulating polymers such as PUR foams are increasingly crucial for reducing building energy losses and overall carbon footprints.

Recent advances in polyurethane research have focused strongly on implementing green technologies and renewable raw materials. Considerable scientific attention has been directed toward the synthesis of polyols derived from non-edible vegetable oils, including camelina, waste rapeseed oil, or fruit seed oils, which can effectively substitute petrochemical feedstock in the production of open-cell foams [5,6,7,8,9]. Another renewable source gaining interest is tall oil, a by-product of cellulose pulping, used as a sustainable feedstock for “green” polyol synthesis [11,12,13,14,15]. The chemical modification of such oils typically involves epoxidation of unsaturated fatty acid double bonds followed by ring opening of oxirane groups, yielding hydroxyl-functional polyols suitable for PUR formulations. Other established methods include ozonolysis and transesterification, both enabling tailored hydroxyl functionality and viscosity [16,17,18,19,20]. Additionally, bio-based fillers such as chitin or nanocellulose can be incorporated to enhance foam properties, while innovative non-isocyanate polyurethane systems are being explored to reduce toxicity and carbon emissions during synthesis.

Despite significant technological progress, the share of bio-based polyols and isocyanates in the global polyurethane market remains limited—estimated at only 2% and 1%, respectively, in 2024. However, projections suggest these proportions may increase to 34% and 13% by 2034. Consequently, the share of bio-based foams, particularly flexible ones, is expected to rise dramatically—from the current 6% to nearly 60% in the coming decade. These trends clearly demonstrate the accelerating shift toward the use of renewable resources in polyurethane foam production and the growing importance of sustainable materials in modern polymer chemistry.

Current environmental regulations have prompted an increased demand for environmentally friendly alternatives. Consequently, significant research efforts in both academia and industry have focused on developing methods for producing polyols from renewable biomass sources (biopolyols). A wide range of sustainable feedstocks—such as vegetable oils, microalgae, lignocellulosic materials, and polysaccharides—have been explored for their potential use in polyol synthesis [21,22,23].

The innovative aspect of this study lies in the strategic selection and combination of two renewable polyol sources—oilseed radish and tall oil—for the synthesis of open-cell polyurethane foams. Oilseed radish polyol represents an emerging class of bio-polyols obtained from non-edible vegetable oils, offering a sustainable alternative that avoids competition with food resources, while tall oil is an abundant by-product of the cellulose pulping industry and one of the most industrially relevant bio-based feedstocks in Northern Europe. Although both polyols have individually demonstrated potential for polyurethane production, their complementary chemical characteristics—particularly the lower hydroxyl number and higher molecular weight of radish polyol versus the higher functionality and reactivity of tall-oil-derived polyols—create an opportunity for synergistic tuning of foam morphology and performance. To date, no systematic investigation has examined how blending these two bio-polyols influences foam reactivity, cellular structure, and mechanical–thermal properties. This study fills that gap by providing the first comparative evaluation of mixed bio-polyol systems, thereby advancing the development of eco-efficient polyurethane materials. The resulting foams show promising potential for use as sustainable thermal insulation and as bio-based adhesives in mineral wool sandwich panel applications.

## 2. Experimental Part

### 2.1. Manufacturing of Open-Cell Polyurethane Foams

**Figure 1:**
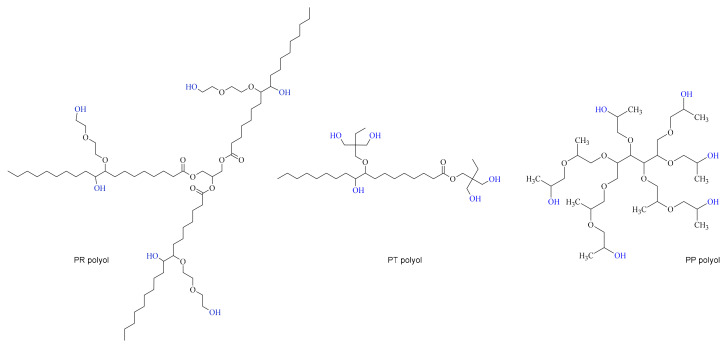
Idealized chemical structure of PR, PT and PP polyols.

**Figure 2:**
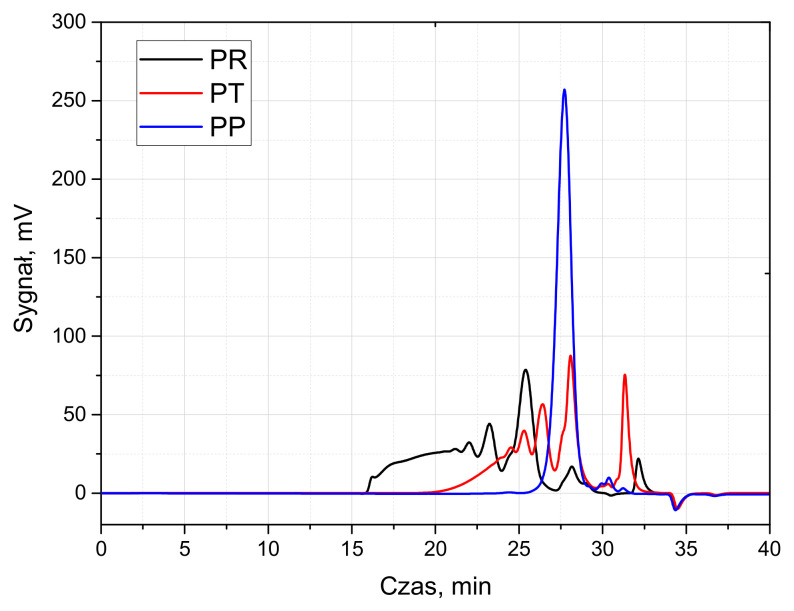
GPC chromatograms of polyols.

Three types of polyols were used to produce PUR foams: (1) the petrochemical polyol Lupranol 3422 (BASF, Lemförde, Germany), (2) a bio-based tall oil polyol synthesized at the Latvian State Institute of Wood Chemistry (LSIWC, Riga, Latvia) from tall oil fatty acids (Forchem Oyj, Rauma, Finland) through epoxidation of the double bonds, followed by oxirane ring opening and esterification of the carboxylic acid groups with trimethylolpro-pane (Sigma-Aldrich, Schnelldorf, Germany), and (3) a bio-based oilseed radish polyol synthesized at the Department of Polymer Chemistry and Technology, Cracow University of Technology, by epoxidation of the oil and subsequent oxirane ring opening with dieth-ylene glycol (Sigma-Aldrich, Schnelldorf, Germany). The idealized chemical structure of the polyols is depicted in Figure 1. The polyols’ characteristics are presented in Table 1 and Gel Permeation Chromatography (GPC) chromatograms are shown in Figure 2.

Catalysts and surfactants supplied by Momentive Performance Materials GmbH Sp z o.o. (Kędzierzyn-Koźle, Poland) were also used to prepare open-cell polyurethane foams. Polymeric diphenylmethane 4,4′-diisocyanate (pMDI) with 31 wt.% free isocyanate groups was supplied by BorsodChem Zrt. (Kazincbarcika, Hungary) The flame retardant tris(1-chloro-2-propyl) phosphate (TCPP) was supplied by LANXESS AG (Cologne, Germany).

Open-cell PUR foams were produced using a single-stage method using two components (A and B). Component A, known as the polyol masterbatch, consists of polyol(s) in specific mass ratios, surfactants, catalysts, water, and flame retardants. Component B is an isocyanate. Initially, the constituents of the polyol premix were mixed for 15 s, after which component B was added and the system was mixed for an additional 5 s. The resulting reactive mixture was poured into an open mold, where free-rise foaming proceeded in the vertical direction. After curing, the biofoams were cut into specimens of the required dimensions.The quantities of the components used are presented in Table 2.

### 2.2. Research Methodology

*Hydroxyl number (OHv)*—was determined using a pyridine-free titration method. This method is based on the acetylation of hydroxyl groups with pyromellitic anhydride dissolved in acetone, using 1-methylimidazole as a catalyst. After the reaction, the excess anhydride is hydrolyzed with water, and the formed acids are titrated with a standardized sodium hydroxide solution in the presence of thymolphthalein in ethanol as an indicator. The hydroxyl number was calculated according to the following equation:

(1) $$OHv=\frac{\left(V_{1}-V_{2}\right)\cdot C\cdot 56.11}{m}\left[\frac{mgKOH}{g}\right]$$

where *V*_1_—volume of NaOH solution used for titration of the blank, cm^3^; *V*_2_—volume of NaOH solution used for titration of the sample, cm^3^; *C*—concentration of NaOH solution, mol/dm^3^; 56.1—molar mass of KOH, g/mol; *m*—sample mass, g.

*Water content* was determined using the Karl Fischer titration method in accordance with PN-81/C-04959, employing a TitroLine TA 05 Plus instrument (SCHOTT Instruments GmbH, Mainz, Mainz, Germany) [24]. The method is based on the quantitative reaction between water and Karl Fischer reagent, with the equivalence point determined by volumetric titration.

*Gel permeation chromatography (GPC)* was used to determine the average molecular weights of oils and biopolyols. An Azura^®^ chromatograph from Knauer (Berlin, Germany) was equipped with thermostated columns and a refractometric detector. The analysis was performed at 35 °C using tetrahydrofuran as the eluent at a flow rate of 1 cm^3^/min. The number-average molar masses (Mn), weight-average molar masses (Mw) and dispersity (D) were determined using calibration with standard polystyrene standards.

*Foaming process analysis*—The foaming process was analyzed using a FOAMAT^®^ system (Format Messtechnik GmbH, Karlsruhe, Germany). The device includes a computer, laboratory scale, mechanical stirrer, ultrasonic sensor, foam pressure sensor, dielectric polarization sensor, and thermocouple. During the foaming reaction, temperature, internal pressure, dielectric polarization, weight loss, and foam height were continuously recorded. The polyol premix was mixed with isocyanate and poured into a measuring tube, with the thermocouple inserted into a predrilled cavity.

*Apparent density*—ISO 845—Foam samples (approx. 20 cm × 20 cm × 5 cm) were cut, and their dimensions were measured using a caliper [25]. Weight was determined on a precision balance. Apparent density was calculated as mass divided by volume, averaged over three specimens of identical formulation.

The morphology of cells was analyzed using a scanning electron microscope TM3000 (Hitachi, Tokyo, Japan). Foam samples of 1 cm × 1 cm × 1 cm before observation were covered with gold using a Polaron SC7640 duster (Polaron Equipment Ltd., Watford, UK). The sputtering process was carried out for 90 s at a current of 10 mA. Observations were carried out at an accelerating voltage of 15 keV. Microphotographs of the cellular structure in the direction perpendicular to the direction of foam rise were made using also an optical microscope (PZO, Warsaw, Poland) equipped with a camera. Then, microphotographs were analyzed regarding the cell size, cell density and anisotropy index using Aphelion software. The ratio of the average height and width of the cells in the cross-sections of tested foams allowed for the calculation of the cell anisotropy index.

*Closed-cell content*—ISO 4590—Foam samples (approx. 3 cm × 3 cm × 10 cm) were prepared and their dimensions measured [26]. The closed-cell content was calculated according to ISO 4590, based on the arithmetic mean from four specimens for each formulation.

*Thermal conductivity (λ)*—The thermal conductivity coefficient (λ) was determined using a Laser-Comp FOX 200 analyzer (TA Instruments, New Castle, DE, USA). The temperature difference between the hot and cold plates was 20 °C. Samples measuring 20 cm × 20 cm × 5 cm (same as in density test) were used. Each test included 8–12 measurements, and λ was reported as an average of two samples per formulation.

*Compressive strength*—ISO 844—The compressive strength was determined with a Zwick Z005 TH testing machine (Zwick GmbH & Co., Ulm, Germany) [27]. Eight cylindrical specimens (≈ 4 cm diameter × 4 cm height) were tested—four parallel and four perpendicular to the foam rise direction—at 10% deformation. Sample dimensions were measured precisely and entered into the control software prior to testing.

*Thermogravimetric analysis (TGA)* was carried out using a Discovery TGA system (TA Instruments, New Castle, USA). PIR foam samples were heated at a rate of 10 °C/min under a nitrogen atmosphere from 30 °C to 700 °C, using platinum pans.

*Dimensional stability under specified temperature conditions*—PN-EN 1604+AC—Samples (100 mm × 100 mm × 25 mm) were measured in three points for length and width, and in four points for thickness [28]. Initial dimensions (a_0_, b_0_, c_0_) were recorded, then samples were placed in a chamber at 70 °C for 24 h, followed by re-measurement after stabilization at room temperature. The same procedure was applied at –20 °C. Dimensional changes were calculated as:

Δ*ε*_a_ = (a_1_ − a_0_)/a_0_ × 100% (2)

Δ*ε*_b_ = (b_1_ − b_0_)/b_0_ × 100% (3)

Δ*ε*_c_ = (c_1_ − c_0_)/c_0_ × 100% (4)

where a_1_, b_1_, c_1_ are final dimensions after stabilization.

## 3. Results and Discussion

**Figure 3:**
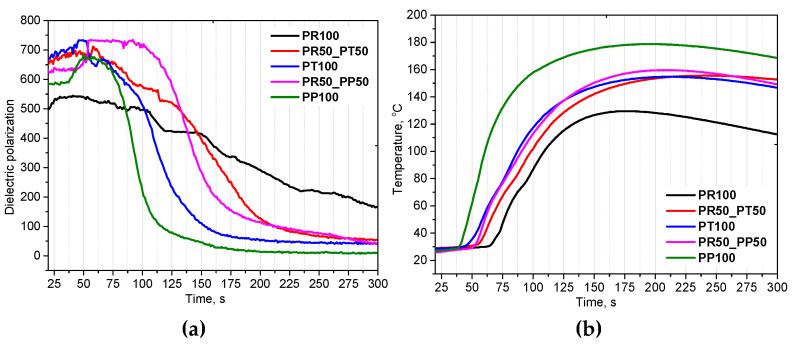
Influence of the type of biopolyol and its content on the dielectric polarization (**a**) and temperature (**b**) during the foaming process.

The foaming process was investigated using the FOAMAT device, which allows for the determination of characteristic parameters during foaming of PUR systems, such as dielectric polarization, height, and temperature. Dielectric polarization reflects the degree of functional group conversion during PUR formation. Figure 3 shows the dependence of these parameters on time.

Based on the reduction in dielectric polarization of the reaction, it was found that the reactivity of systems modified with the oilseed radish biopolyol was lower than that of foams made with tall oil biopolyol and petrochemical polyol. The PUR formation reactions are highly exothermic. The rate of temperature increase determines the reactivity of PUR systems. All systems modified with various mass fractions of oilseed radish biopolyol were characterized by a lower maximum temperature than those without the oilseed radish biopolyol. The maximum temperature during the foaming reaction was the lowest for the system composed of oilseed radish biopolyol (PR100) and the highest for the petrochemical polyol (PP100). In the case of the PT100 system, despite its high reactivity, the temperature inside the sample was comparable to that of the systems modified with PR biopolyol. This is advantageous in terms of the possibility of sample burn-through caused by high temperatures. The lowest reactivity of the biopolyol may be related to its lowest hydroxyl number among the polyols used. In previous studies it was investigated the effect of rapeseed oil-based polyols on the foaming process of rigid polyurethane foams. They obtained a similar effect of reducing the maximum temperature during the foaming reaction with increasing bio-polyol content. The maximum temperature during foaming of the reference system was similar, approximately 170 °C. The maximum temperature of the reaction mixture during foaming was reduced to approximately 150 °C when the petrochemical polyol was replaced with a rapeseed oil-based polyol in the amount of 70% by weight [29]. The high reactivity of bio-based polyols offers a significant advantage in formulation development, as it enables a reduction in catalyst concentration while maintaining the same reactivity level as petrochemical counterparts. Lower catalyst content not only decreases material costs but also minimizes the release of volatile organic compounds (VOCs) during processing and curing [30]. This contributes to a more environmentally friendly and sustainable production process, while preserving the mechanical and adhesive performance required for high-quality polyurethane materials.

**Figure 4:**
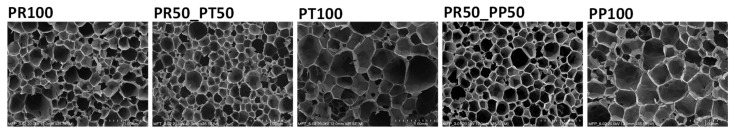
SEM images of the obtained open-cell PUR foams in the direction perpendicular to the growth of the foam material.

In porous materials, cellular structure plays a significant role. The shape of the cells influences both thermal insulation properties and mechanical strength. Cellular structure analysis was performed using scanning electron microscopy and optical microscopy. Figure 4 shows SEM images of the tested PUR materials.

**Figure 5:**
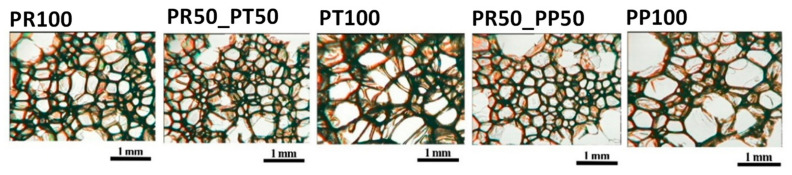
Cell structure of open-cell PUR foams in the direction perpendicular to the foam growth.

It was observed that the highest cell density was obtained in the case of a mixture of radish biopolyol with tall oil polyol, as well as with a petrochemical polyol. This may indicate that the lightness of the polyol plays a significant role in this process, rather than necessarily the chemical structure of the polyol. This opens up a wide range of possibilities for using various raw materials, including those derived from biomass. Tall oil biopolyol foams modified with radish biopolyol are characterized by smaller pores than the unmodified foam (PT100). Similar relationships are observed for petrochemical polyol foams modified with radish biopolyol. Foams modified with radish biopolyol are characterized by finer pores than those made with petrochemical polyol (PP100). The effect of biopolyols on the cell structure of foams is in Figure 5 and Table 3.

The use of petrochemical polyol or bio-based tall oil polyol with bio-based oilseed radish polyol does not produce significant differences in the resulting polyurethane materials. This demonstrates the versatility of using bio-based oilseed radish polyol compared to other polyols. All foams tested perpendicularly have a cell shape that is close to isotropic, as their anisotropy coefficient is close to 1.

**Figure 6:**
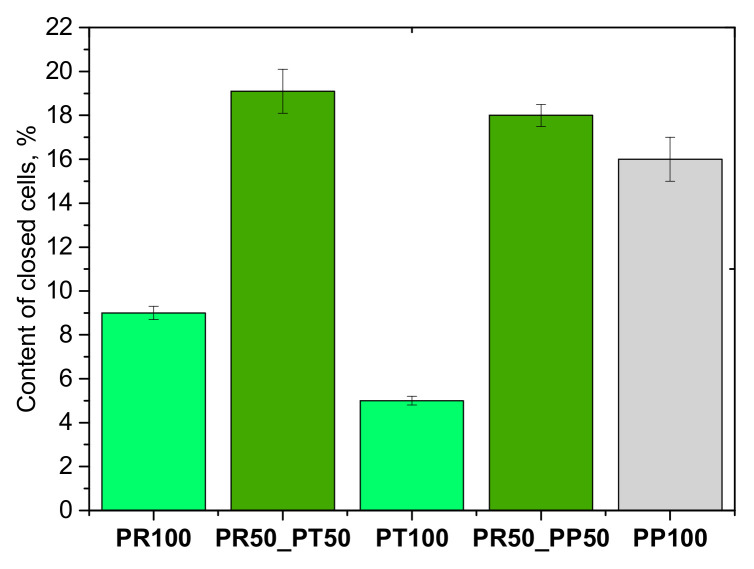
Closed cell content of open-cell PUR foams.

Open-cell foams generally have a low closed-cell content (<20%). Thermal conductivity and compressive strength, among other things, depend on the closed-cell content. Figure 6 shows the closed-cell content of the tested PUR materials.

**Figure 7:**
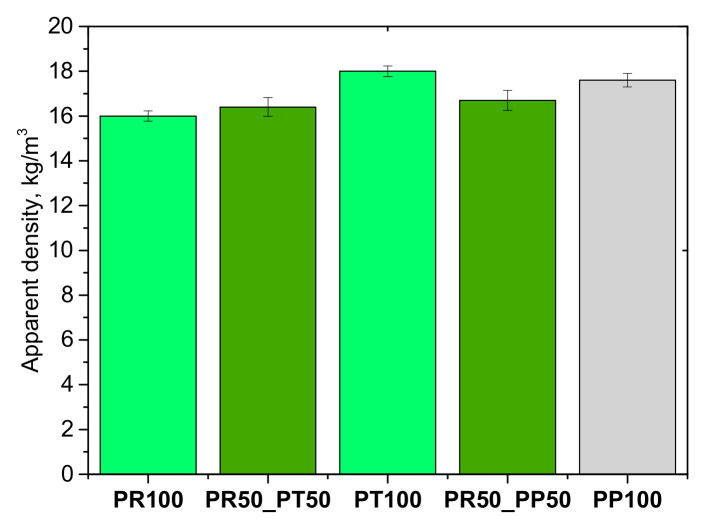
Apparent density of open-cell PUR foams.

It was observed that in the case of using mixtures of biopolyols and foam produced from petrochemical biopolyol, the content of closed cells was slightly higher than in the case of foams made of 100% biopolyols PR and PT. Apparent density is one of the most important parameters characterizing polyurethane foams. Figure 7 presents the apparent density values of foams containing biopolyol from oilseed radish, tall oil and petrochemical polyol.

The average apparent densities of the obtained open-cell polyurethane foams ranged from 16 to 18 kg/m^3^. Comparable results were reported by K. Polaczek et al., who produced open-cell foams by partially substituting a petrochemical polyol with a palm oil–based bio-polyol (20 wt.%), obtaining apparent densities between 13 and 18 kg/m^3^ [31]. In the present study, the measured densities exhibited uniform distribution across sampling points, as indicated by low standard deviations. The highest apparent densities were recorded for systems formulated with 100% petrochemical polyol and 100% tall oil–derived bio-polyol, while the lowest density was observed for foams in which 50% of the petrochemical polyol was replaced with bio-based oilseed radish polyol. A consistent trend of decreasing apparent density was observed with increasing content of the oilseed radish bio-polyol. This reduction can be attributed to its lower hydroxyl value compared to both the petrochemical and tall oil–based polyols, which influences crosslinking density and gas retention during foaming. The low apparent density of these polyurethane foams is advantageous for the development of cost-effective thermal insulation materials and particularly beneficial for use as high-performance adhesives in mineral wool sandwich panels, as the reacting foam can readily penetrate the porous mineral wool structure and form a strong, uniform adhesion layer.

**Figure 8:**
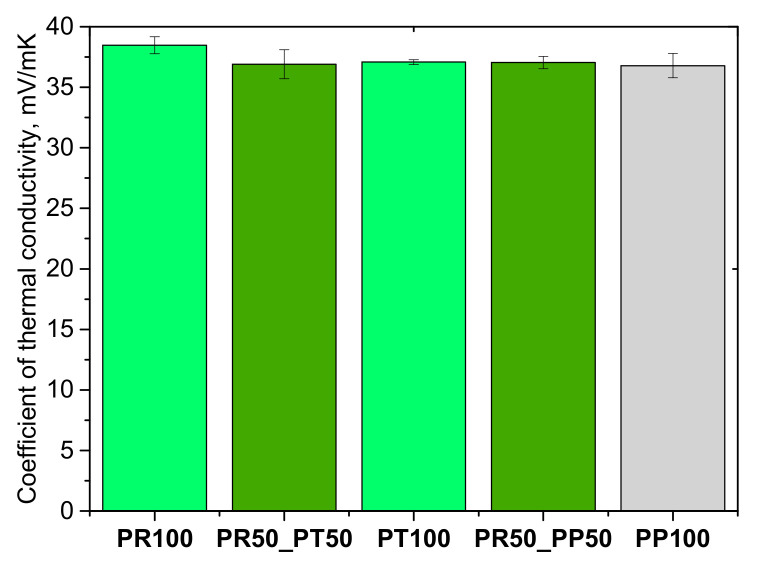
Coefficient of thermal conductivity of open-cell PUR foams.

The thermal conductivity of foams is an important property, partly because PUR foams are currently considered one of the best insulating materials. The obtained test results are presented in Figure 8.

Open-cell PUR foams exhibit considerably higher thermal conductivity than closed-cell foams. This results from their open-cell morphology, where the blowing agent is rapidly replaced by air, yielding thermal conductivity values of approximately 0.037–0.039 W/m·K. In contrast, closed-cell foams typically show much lower thermal conductivity, around 0.018–0.030 W/m·K, largely determined by the composition of the gas trapped within the sealed cells.

Modification of polyurethane systems with bio-based polyols and petrochemical polyols at had a minor effect on the thermal conductivity coefficient. Values ranged from 36 to 39 mW/m∙K. The highest thermal conductivity coefficient was observed for the foam obtained from the bio-based polyol derived from oilseed radish (100% by weight), at 38.5 mW/m∙K.

**Figure 9:**
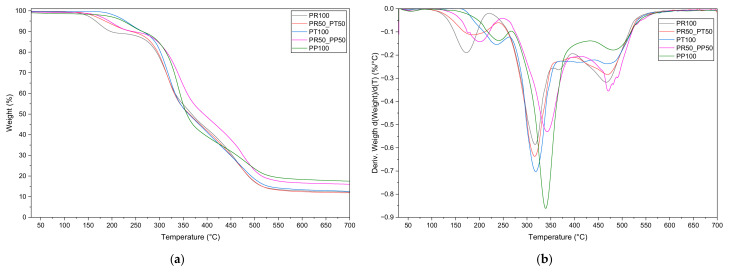
TG (**a**) and DTG (**b**) curves of open-cell foams.

Thermogravimetric analysis was conducted to evaluate the thermal stability and decomposition behavior of the synthesized polyurethane foams. The mass loss graph and derivative of the mass loss is depicted in Figure 9. And the analysis of the main degradation steps is summarized in Table 4. The analysis revealed a characteristic two-step degradation pattern typical for urethane-based materials. The initial minor weight loss below 200 °C was attributed to the evaporation of residual moisture and volatile compounds trapped in the foam structure, such as flame retardant. The first major degradation stage, occurring between 200 °C and 350 °C, corresponded to the thermal cleavage of urethane bonds and decomposition of hard segments, while the second stage, between 350 °C and 500 °C, was associated with the breakdown of soft polyol segments through ether and ester bond scission. Above 500 °C, the formation of a stable carbonaceous residue was observed, indicating char formation and the final degradation stage. Samples containing mixed or bio-based polyols exhibited slightly higher onset degradation temperatures and greater residual mass at 700 °C, confirming enhanced thermal stability and char-forming ability compared to fully petrochemical systems. These results highlight the potential of bio-polyol formulations to improve flame resistance and thermal endurance of polyurethane materials.

Overall, the TGA results confirm that the developed PUF formulations possess notable char-forming ability—a critical property for achieving high-performance adhesives and thermally stable insulation materials used in mineral wool sandwich panel applications.

**Figure 10:**
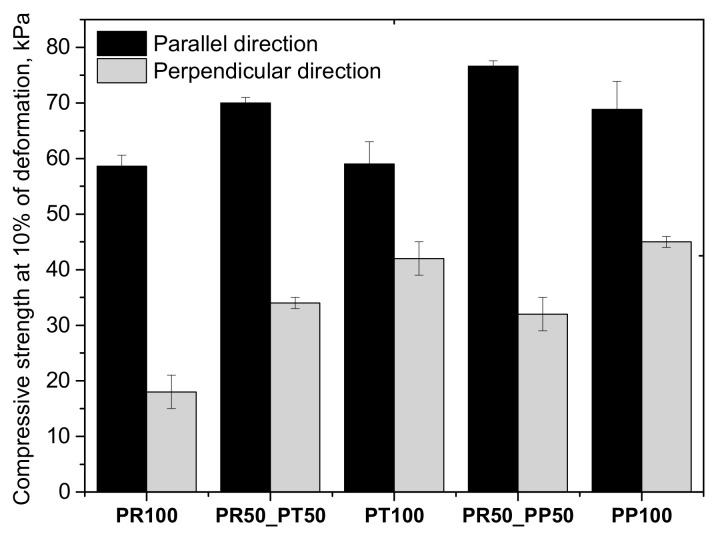
Compressive strength of open-cell PUR foams.

Compressive strength depends on the cellular structure of the material and its apparent density. The compressive strength test results are shown in Figure 10.

Compressive strength is significantly higher in the direction parallel to the foam growth than in the perpendicular direction. This difference may be due to the anisotropic nature of PUR foams. The highest compressive strength values in the direction parallel to the foam growth direction are achieved by foams with the highest closed-cell content. The compression strength correlates with the cell density results depicted in Table 3. The highest compression strength of 75 ± 2 kPa was for PU foams with a cell density of 5.95 ± 0.72 cm^3^·10^5^. This indicates that the closed-cell content influences the mechanical strength of PUR foams. Dimensional stability depends, among others, on mechanical strength. Dimensional stability measurements of the obtained PUR foams depending on different shares of petrochemical polyol, tall oil bio-polyol and oil radish bio-polyol are summarized in Table 5 and Table 6.

The obtained average dimensional stability values were less than 0.5% regardless of the measurement direction. Based on this, it can be seen that replacing the petrochemical polyol and the tall oil biopolyol with the radish biopolyol did not significantly affect the stability of the resulting open-cell foams. All obtained PUR foams were characterized by good dimensional stability at both high and low temperatures. The radish biopolyol can be used in a polyurethane system with both the petrochemical polyol and the tall oil biopolyol, as no significant differences were observed in the resulting foam materials.

When compared with polymeric foams described in the literature, the developed bio-based open-cell polyurethane foams exhibit performance characteristics that fall within the expected property window for conventional open-cell PUR and other bio-polyol–based foams. Their apparent density (15–18 kg/m^3^) and thermal conductivity (36–39 mW/m·K) [32] closely match the values reported for petrochemical and vegeta-ble-oil-derived open-cell foams, confirming that the partial or full substitution of petro-chemical polyols does not compromise insulation performance [33]. Mechanical properties and dimensional stability likewise remain comparable to commercial systems, with com-pressive strength values typical for anisotropic open-cell PUR structures and dimensional changes below 0.5% [34,35]. The Nia S. and Nikje M. have reported compression strength of bio-based PU foams of 0.05 to 0.22 MPa, which is similar to results presented in this work [35]. A notable advantage of the new formulations is their improved thermal stability and higher char residue, which exceed those of petrochemical references and are con-sistent with literature trends for tall-oil- and seed-oil-based polyurethanes, suggesting en-hanced fire resistance potential [36]. Additional benefits include reduced dependence on fossil resources, lower expected VOC emissions due to reduced catalyst demand, and the ability of low-density foams to penetrate porous substrates such as mineral wool, making them suitable for bio-based adhesives. The main limitations relate to slightly reduced reac-tivity and marginally higher λ-values at very high contents of oilseed radish polyol, which may require formulation adjustments (e.g., optimized catalysts or polyol ratios). Overall, the results demonstrate that the new bio-based foams are competitive with established polymeric foams while offering meaningful sustainability advantages.

## 4. Conclusions

Based on the conducted study of open-cell polyurethane foams prepared using oilseed radish bio-polyol, petrochemical polyol, and tall oil bio-polyol, it was found that the incorporation of renewable components significantly influenced the foaming behavior, cellular morphology, and resulting physical–mechanical properties of the materials. FOAMAT analysis indicated that systems containing oilseed radish bio-polyol exhibited lower reactivity compared to petrochemical and tall oil-based systems, as evidenced by a lower maximum reaction temperature and a slower decline in dielectric polarization.

The apparent densities of the obtained foams ranged between 15 and 18 kg/m^3^. An increase in the oilseed radish bio-polyol content caused a slight decrease in density, which can be attributed to its lower hydroxyl value relative to petrochemical and tall oil-derived polyols. The thermal conductivity coefficients of the foams were within the range of 36–39 mW/m·K, consistent with values typical for open-cell polyurethane materials. A minor increase in thermal conductivity was observed as the proportion of oilseed radish bio-polyol increased. The compressive strength was higher in the direction parallel to the foam growth—characteristic of anisotropic open-cell foams—and the highest values were achieved for samples with a larger proportion of closed cells. All foams demonstrated excellent dimensional stability, with volume changes below 0.5% at both elevated (70 °C) and sub-ambient (–20 °C) conditions. Notably, the partial or complete substitution of petrochemical and tall oil-based polyols with oilseed radish bio-polyol did not impair mechanical integrity or dimensional stability.

In conclusion, the results confirm that oilseed radish bio-polyol is a viable renewable alternative to conventional petrochemical and tall oil polyols for producing open-cell polyurethane foams. The developed formulations maintain favorable mechanical, thermal, and structural performance while reducing dependence on fossil-based raw materials. These bio-based foams represent an important step toward sustainable polyurethane systems, with potential applications not only in thermal insulation but also as high-performance adhesives for bonding mineral wool cores in sandwich panel production.

## Figures and Tables

**Table 1:** Selected properties of bio-polyols.

Polyol		OHv, mgKOH/g	%H_2_O, mas.	Mn, g/mol	Mw, g/mol	D
PR	Bio-polyol from oil radish	167	0.05	1135	4126	3.64
PT	Bio-polyol from tall oil	406	0.13	448	910	2.03
PP	Petrochemical polyol	476	0.11	482	506	1.05

OHv—hydroxyl value; %H_2_O—water content; Mn—number-average molar masses; Mw—weight-average molar masses; D—dispersity.

**Table 2:** PUR foam formulations.

Component	PR100	PR50_PT50	PT100	PR50_PP50	PP100
PR	100	50	0	50	0
PT	0	50	100	0	0
PP	0	0	0	50	100
Flame retardant	40	40	40	40	40
Water	15	15	15	15	15
Catalyst 1	0.5	0.5	0.5	0.5	0.5
Catalyst 2	3	3	3	3	3
Catalyst 3	0.2	0.2	0.2	0.2	0.2
Surfactant 1	0.5	0.5	0.5	0.5	0.5
Surfactant 2	5	5	5	5	5
pMDI	262.1	290.3	318.4	298.7	335.5

In this paper, the symbols for individual PUR foams are assigned as follows: PP—petrochemical polyol, PR—biobased polyol from oilseed radish, PT—biobased polyol from tall oil. The numbers after PP, PR, and PT indicate the mass ratio of the added polyols. For example, PR50_PP50 foam contains 50 g of petrochemical polyol and 50 g of bio-based polyol from oilseed radish.

**Table 3:** Characterization of the cell structure of open-cell PUR foams in the direction perpendicular to the foam growth.

PUF Name	Anisotropy Index	$\mathrm{Cell}\;\mathrm{Density},\;\mathrm{Cell}\;\mathrm{Count}\;\mathrm{in}\;{\mathrm{cm}}^{3}\cdot$10^5^
PR100_p	0.87 ± 0.04	4.65 ± 0.51
PR50_PT50_p	1.04 ± 0.07	5.04 ± 0.81
PT100_p	0.90 ± 0.11	4.40 ± 0.64
PR50_PP50_p	0.91 ± 0.06	5.95 ± 0.72
PP100_p	0.90 ± 0.05	3.96 ± 1.26

**Table 4:** TGA of the open-cell PUR foams.

Sample	Onset Degradation (°C)	Main Peak (°C)	Residue @ 700 °C (%)
PR100	~250	~300	~10
PR50_PT50	~260	~305	~14
PT100	~270	~310	~15–18
PR50_PP50	~290	~320	~20
PP100	~240	~340	~20

**Table 5:** Dimensional stability of open-cell PUR foams measured at 70 °C.

PUF Name	$∆\varepsilon_{a}$	$∆\varepsilon_{b}$	$∆\varepsilon_{c}$
PR100	−0.24 ± 0.18	−0.08 ± 0.14	−0.41 ± 0.42
PR50_PT50	−0.24 ± 0.05	−0.06 ± 0.11	−0.49 ± 0.29
PT100	−0.34 ± 0.01	−0.26 ± 0.03	−0.49 ± 0.07
PR50_PP50	−0.16 ± 0.13	−0.04 ± 0.1	−0.45 ± 0.31
PP100	−0.29 ± 0.14	−0.25 ± 0.06	−0.53 ± 0.03

**Table 6:** Dimensional stability of open-cell PUR foams measured at 20 °C.

PUF Name	$∆\varepsilon_{a}$	$∆\varepsilon_{b}$	$∆\varepsilon_{c}$
PR100	0.43 ± 0.08	0.36 ± 0.13	0.18 ± 0.17
PR50_PT50	0.20 ± 0.11	0.15 ± 0.04	−0.26 ± 0.22
PT100	0.03 ± 0.23	−0.08 ± 0.23	0.04 ± 0.28
PR50_PP50	0.20 ± 0.02	0.08 ± 0.03	−0.03 ± 0.42
PP100	0.10 ± 0.05	0.06 ± 0.002	−0.17 ± 0.11

## Data Availability

The original contributions presented in this study are included in the article. Further inquiries can be directed to the corresponding authors.
